# Hypoalgesic and Motor Effects of Neural Mobilisation versus Soft-Tissue Interventions in Experimental Craniofacial Hyperalgesia: A Single-Blinded Randomised Controlled Trial

**DOI:** 10.3390/jcm10194434

**Published:** 2021-09-27

**Authors:** Marta Díaz-Sáez, Cristina Sáenz-Jiménez, Jorge Hugo Villafañe, Alba Paris-Alemany, Roy La Touche

**Affiliations:** 1 Centro Superior de Estudios Universitarios La Salle, Departamento de Fisioterapia, Universidad Autónoma de Madrid, 28023 Madrid, Spain; madisa@irflasalle.es (M.D.-S.); csaeji@campuslasalle.es (C.S.-J.); roylatouche@yahoo.es (R.L.T.); 2 IRCCS Fondazione Don Carlo Gnocchi, 20148 Milan, Italy; mail@villafane.it; 3 Centro Superior de Estudios Universitarios La Salle, Motion in Brains Research Group, Institute of Neuroscience of the Movement (INCIMOV), Universidad Autónoma de Madrid, 28023 Madrid, Spain; 4 Instituto de Dolor Craneofacial y Neuromusculoesquelético (INDCRAN), 28008 Madrid, Spain

**Keywords:** myofascial trigger points, trigeminal nerve, neurodynamics, neural mobilisation, soft-tissue, manual therapy, pressure pain threshold, provocation chewing test

## Abstract

Objective: The present trial aimed to compare the effects of the mobilisation of the nervous system (NS) to those of a soft-tissue intervention in subjects exposed to an experimentally induced hyperalgesia of the masticatory muscles. Methods: The study was a single-blinded randomised controlled trial. A total of 49 participants (mean ± SD age: 41 ± 11 years; 61% female) with latent myofascial trigger points (LMTrPs) in the craniofacial region were randomly assigned to one of three groups: neural mobilisation (NM), soft-tissues techniques and stretching (STT-S), and control group (CG). An initial assessment (baseline) was performed before the provocation chewing masticatory test. The pre-treatment measurements were registered 24 h later. Next, the randomised intervention was applied, and afterwards, post-treatment data were obtained. Outcome measures included pain-free maximum mouth opening (MMO), pressure pain thresholds (PPTs) in the trigeminal and cervical region, and trigeminal and cervical two-point discrimination (TPD). Results: ANOVA revealed significant differences for the time × group interaction for pain-free MMO and PPTs. The results showed an improvement in the MMO and the PPTs for NM and STT-S groups but not for the CG. There were no differences between the NM and STT-S groups. However, the effect sizes were large for the NM and medium for the STT-S. No differences were found for TDP between groups nor over time. Conclusions: The results show that with NM and STT-S techniques, we could influence motor and sensory variables in asymptomatic subjects with LMTrPs after a masticatory provocation test. Both techniques increased MMO and PPTs in the short term. These beneficial effects lead us to consider the importance of including these methods in clinical practice.

## 1. Introduction

Latent myofascial trigger points (LMTrPs) are subclinical neuromuscular lesions common in healthy subjects and in patients with musculoskeletal pain. According to Lucas et al. [1] 89.8% of 137 subjects presented at least one LMTrP. A LMTrP is defined as a focus of hyperirritability in a taut muscle band that is clinically associated with local twitch response and tenderness and/or referred pain upon manual examination [2]. Li et al. have found that LMTrPs are loci that present with a nociceptive hypersensitivity [3]. They show the same clinical characteristics as active myofascial trigger points (MTrPs), except they do not provoke spontaneous pain [2].

MTrPs have been associated with sensory disturbances and motor dysfunctions [2,4,5]. Specifically, LMTrPs can produce increased muscle tension and shortening [6], restricted range of movement [7,8,9], muscle weakness [2,10,11], muscle fatigue [11], alternating muscle activation patterns, and cramping [12,13,14]. It has been demonstrated that the prolonged stimuli over a LMTrP may lead to an active MTrP. Prolonged exercises, low-load but repetitive exercises or movements, persistent muscle stress, and prolonged ischemia are examples of the persistent stimuli associated with active MTrP and LMTrP [15,16]. Hence, when a muscle presenting LMTrPs becomes overloaded mechanically, the subject could end up experiencing pain [1]. Several studies have demonstrated the clinical relevance of LMTrPs, particularly related to muscle function and pain sensitisation mechanisms [2,4,5].

Regarding the craniofacial area, the presence of LMTrPs in the masticatory muscles has been found together with reduced jaw opening [17]. Several studies have used individuals with LMTrPs in the masseter muscle to assess the effects of soft-tissue techniques usually applied to the MTrP, such as sustained pressure, neuromuscular approach, or post-isometric relaxation technique [18,19]. Using soft-tissue techniques can improve the sensitivity of the masticatory LMTrPs (masseter and temporalis) and mouth opening [18].

On the other hand, neural mobilisation (NM) methods have also been used as a clinical tool for the treatment of neuromuscular disorders. This technique aims to facilitate nerve gliding, reduce nerve adherence, and disperse intraneural oedema [20,21]. Boyd et al. [22] observed that the mechanosensitivity of the nervous system (NS) is found together with an increase in muscle tone and a subsequent reduction of the range of motion (ROM) and the onset of symptoms. In this line, neurodynamics is a manual therapy technique used by physical therapists for the evaluation and treatment of neuromuscular disorders [23]. They are characterised by NM that include tension and glide techniques [24]. Those techniques are used frequently in various regions of the body because of the benefits that have been scientifically demonstrated [25,26,27,28]. Beneciuk et al. showed the existence of differences in the temporal summation, as well as the range of motion and sensory descriptors after three weeks using the technique of NM in the upper extremity [29]. The benefits after the NM techniques have been described as improved mobility, reduced sensitivity, and reduced pain [28,30].

While there are many studies demonstrating the efficacy of soft-tissue techniques in LMTrPs or myofascial masticatory pain [17,18,31], there is no literature that compares the effects of mobilisation techniques of NS performed at the craniofacial region. However, it would be interesting to assess this since they have been proven effective at other anatomical segments [25,32]. Along with the idea of assessing craniofacial NM in the present research, an experimental chewing test was used to reach masticatory hyperalgesia [33,34]. The aim of that masticatory provocation test was to evoke experimentally delayed-onset muscle soreness (DOMS) and short-lasting hyperalgesia through a repetitive exercise, which may increase the sensitivity of the craniofacial LMTrPs [35,36,37].

Based on the above, this randomised controlled trial aimed to compare the effect generated by the mobilisation of the NS and soft-tissue techniques on sensory-motor trigeminal variables in the craniofacial and cervical regions of asymptomatic subjects with LMTrP, to whom masticatory hyperalgesia was experimentally induced with a provocation masticatory test.

## 2. Materials and Methods

### 2.1. Study Design

The study was a single-blinded randomised controlled trial. Informed consent was obtained from all participants, and procedures were conducted according to the Declaration of Helsinki. The protocol was approved by the Local Ethical Committee (CSEULS-PI-069/2015) and was registered with ClinicalTrials.gov (NCT03327545). The study was registered as a trial at the Current Controlled Trials website. The present document was prepared according to the CONSORT publishing guidelines [38]. The study was publicly advertised so that people interested in the trial could participate.

### 2.2. Randomisation and Blinding

After meeting the eligibility criteria for the study, participants were randomly allocated by Investigator B to one of the three groups according to the computer-generated random allocation list. Investigator B, who did not participate in data collection from subjects to ensure that the allocation was concealed, kept the randomised list.

The blinding of the study was accomplished through the blinding of Investigator A. Investigator A was blinded to the interventions until the end of the entire data collection period. In addition, participants were instructed not to discuss their interventions with Investigator A.

### 2.3. Selection and Description of Participants

Subjects between the ages of 18 and 65 from the local community of Madrid with LMTrPs in the craniofacial region were recruited into the study from November 2015 to May 2016. Subjects were included if they met all of the following criteria: age in the specified range; no experience of craniofacial, neck or upper limb pain; LMTrPs in the masticatory muscles; complete dentition; no significant history of chronic pain disorder; not using any medication and able to understand, write and speak Spanish and English fluently. The exclusion criteria were craniocervical pain, dental pathology, regular use of chewing gum, peripheral neuropathy, history of migraine, endocrine disorders, epilepsy or any psychiatric disorder, neurological disorder, surgery and a history of traumatic injuries of the upper limb. Each subject signed an informed consent form. Then, participants were randomly allocated to one of three groups: (1) neural mobilisation (NM), (2) soft-tissue techniques and stretching (STT-S) and (3) control group (CG).

### 2.4. Interventions

The three groups attended two sessions with an interval of 24 h between them. On the first day, sensory-motor variables were assessed before (baseline) the provocation chewing test. On the second day, the sensory-motor variables were assessed once again (pre-treatment), followed by the assigned intervention, and afterwards, the final sensory-motor data were collected (post-treatment). The interventions were performed by Investigator B, a physical therapist with more than two years of experience who received previous specific training.

#### 2.4.1. Neural Mobilisation Group

The subjects received treatment with three NM techniques on the right side of the craniocervical region for a total of 12 min (4 min each technique). All treatments were performed with the participant in a supine position (Appendix A).

#### 2.4.2. Soft-Tissue Techniques and Stretching Group

The subjects received STT-S treatment with three different techniques on the right side of the craniocervical region for a total of 12 min. The first technique was based on intermittent compression on some LMTrPs. Then, participants received two stretches of 30 s each. The third technique consisted of 7 min of soft-tissue mobilisation (Appendix B).

#### 2.4.3. Control Group

Participants who were randomly assigned to the control group did not receive any treatment. Investigator B remained in the room with the subject for 12 min to maintain the blinding procedure.

### 2.5. Outcomes and Measurements

Data were collected on socio-demographic variables, such as age, gender, level of education, marital status and employment status, as well as some psychological variables. Apart from socio-demographic variables, some somatosensory and motor variables were also assessed.

#### 2.5.1. Primary Outcome Measurements

*Pressure pain threshold (PPT).* The PPT is defined as the minimum intensity of a stimulus that is perceived as painful [39]. A digital algometer (FDX 25, Wagner, Greenwich, CT, USA), which permits the evaluation of mechanical hyperalgesia, was used to measure this variable [40]. The instrument was applied perpendicular to the skin, and the pressure was gradually increased at a rate of approximately 1 kg/s until the subject commented that the pressure started to change to a pain sensation. At this point, the evaluator stopped applying pressure and recorded the result [41]. The assessment of PPTs was performed for the masseter, temporal, upper trapezius, and suboccipital muscles, all of them at the right side. The anatomical references where the algometer had to be applied were the following: 2.5 cm anterior to the tragus and 1.5 cm inferior to the zygomatic arch, over the superior part of the masseter; 3 cm superior to the line between the lateral edge of the eye and the auricular helix, over the anterior temporalis fibres, middle point between C7 and acromion, over the superior trapezius; area located over suboccipital muscles; based on previously published protocols [42,43]. This measure was performed and registered three times at each point, and the mean obtained was the total value for the statistical analysis. The reliability of pressure algometry was found to be good (intraclass correlation coefficient; ICC: 0.91) [44].

#### 2.5.2. Secondary Outcome Measurements

*Maximum mouth opening (MMO)*. The ability to open the mouth as widely as possible as measured with the craniomandibular scale (CMD. Patent. No is 1075174 U, INDCRAN: 2011. INDCRAN, Madrid, Spain). It is a thin plastic device formed by two parts that permit the evaluation of movements of the temporomandibular joint. The first part has three millimetric rulers, which are used to measure the opening, lateral excursion, and protrusion of the mouth. The second part is a plastic arm attached to the scale with a black line painted on it. This allows measurement of the mandibular deviations. The unit of measure of the scale is millimeters (mm) [45]. The measure was performed with the subject lying supine with a neutral craniocervical position. The subject was given the indication “open your mouth wide open”, then the measure was performed supporting the scale on the left central inferior incisor, and the left central upper incisor offered the data for the MMO. This measure was performed and registered three times, and the mean obtained was the total value for the statistical analysis. The measure of MMO with the CMD obtained good reliability (ICC: 0.95–0.96) [45].

*Two-point discrimination (TPD).* An esthesiometer (Baseline^®^; reference 12-1480, dimensions: 30.48 × 5.08 × 5.08 cm and weight: 0.611 kg) was used to assess tactile acuity in the TPD test. It is a plastic device that consists of two rounded and small tips with a sliding scale calibrated to 0.1 cm. The perpendicular application of the two points over the skin generates the correct tactile stimulus. The two points must be applied at the same time, for 1 s, with enough pressure to allow a slight depression of the skin surface. The TDP was measured over trigeminal and cervical areas. The trigeminal references were forehead, 1 cm lateral to the brow and 2 cm cranial (V1) and cheek, 2 cm lateral from the nasal line and 2 cm caudal to the eye corner (V2). The cervical reference was: the spinous process of C7, performing the measure horizontally out from the midline to the right [46,47]. A starting distance enough for the participant to correctly discriminate was established for each of the measured points: V1, 20 mm, V2, 20 mm, and cervical, 60 mm. Alternating stimuli with increasing and decreasing distance were applied during each series to avoid the expectation of a continuous decrease. The distance was reduced initially by 5 mm, and subsequently, only changes of 1 mm were made to achieve three correct responses of discrimination at the smallest distance. The position of both points was modified for each stimulus. The participants were asked to reply: “one point”, “two points”, or “I don’t know” after each stimulus [46]. The reliability of the TPD test has been considered good (ICC: 0.70–0.86) [46,47].

#### 2.5.3. Psychological Variables

*Pain catastrophising* is the tendency to have a fixation on pain and feel unable to deal with it [48]. It was used in the Spanish version of the pain catastrophising scale (PCS). The PCS has 13 items and a 3-factor structure: rumination, magnification and helplessness. The theoretical range is between 0 and 52, with lower scores indicating less catastrophising. The PCS has demonstrated acceptable psychometric properties [49].

*Fear of movement:* kinesiophobia is defined as an excessive and irrational debilitating fear of physical movement and activity, resulting from a sense of vulnerability due to a painful injury or the possibility of being reinjured [50]. It was assessed using the Spanish version of the Tampa scale for kinesiophobia (TSK-11), which consists of 11 items [51]. The total score range is from 11 to 44, where higher scores indicate greater fear of movement and pain [51].

### 2.6. Provocation Chewing Test

The aim of the provocation chewing test was to generate an experimentally induced hyperalgesia for the participants. This test was performed in a comfortable sitting upright position with the thoracic spine in contact with the back of the chair but without contact with the craniocervical region with the seat. The feet are positioned flat on the floor with knees and hips flexed at 90 degrees and arms resting freely alongside.

The provocation chewing test protocol consisted of 6 min of unilateral chewing of eight grams of hard gum, modified from Karibe et al. [52]. The chewing gum was employed to elicit pain and muscle fatigue. The test was performed using the right side exclusively. A metronome was set at 80 beats per minute to indicate the chewing rate, as documented in a previous study [53]. The participants were instructed to chew gum initially for 60 s to soften it; then, after 70 s of rest, the signal was given to start the chewing test at the right side.

### 2.7. Procedure

Each subject gave informed consent prior to participation in the study. Then, a physical examination was performed by Investigator B to confirm the presence of a LMTrP at the masseter muscle, looking for a local hyperirritable point in a taut muscle band associated with a twitch response tenderness and a distant referred pain upon manual examination [10,54]. Once included and randomised, the participant filled out the socio-demographic, pain catastrophism and fear of movement questionnaires. Following, Investigator A collected the MMO, TPD, and PPT baseline data. Subsequently, the provocation chewing test was performed. Twenty-four hours later, the pre-treatment measurements of MMO, TPD, and PPT were registered by Investigator A, each participant received the assigned intervention for 12 min from Investigator B. In the NM group, participants received three neural mobilisation techniques on the craniocervical region. In the STT-S group, a soft-tissue treatment including stretching was administered by Investigator B. The CG did not receive any treatment. Finally, 5 min after the intervention, the post-treatment variables assessment was performed by Investigator A for MMO, TPD, and PPT after the intervention.

### 2.8. Sample Size Calculation

The sample size and power calculations were performed using software from the Massachusetts General Hospital’s (MGH) Biostatistics Center (Boston, MA). The PPT was chosen as the primary outcome measure in this study. Calculations were based on a minimal detectable change of 1.04 kg/cm^2^ for the trapezius muscle, based on data from a previous study [41], assuming a standard deviation of 1.16 kg/cm^2^, two-tailed tests and an alpha level of 0.5. This generated a sample size of 36 participants for the study to have 90% power to identify an effect. Allowing for a dropout rate of 25%, we planned to recruit at least 45 patients.

### 2.9. Statistical Analysis

Data were analysed using SPSS version 22.0 (IBM Corporation, Armonk, NY, USA). The normality of the variables was evaluated by the Shapiro–Wilk test. Descriptive statistics were used to summarise the data for categorical variables as absolute (number) and relative frequency (percentage), psychological variables are presented as median and interquartile range (25–75%), and age as mean ± standard deviation (SD), 95% confidence interval (CI). One-way ANOVA was used to compare continuous variables. A chi-squared test with residual analysis was used to compare categorical variables. A 2-way repeated-measures ANOVA was used to compare continuous outcome variables. The factors analysed were group (NM group, STT-S group and CG) and time (baseline, pre-treatment and post-treatment). The time × group interaction, which is the hypothesis of interest, was also analysed. The partial eta-squared (*ηp*^2^) was calculated as a measure of effect size (strength of association) for each main effect and interaction in the ANOVAs: 0.01–0.059 represented a small effect, 0.06–0.139 a medium effect and >0.14 a large effect [55]. Post hoc analysis with a Bonferroni correction was performed in the case of significant ANOVA findings for multiple comparisons between variables. Effect sizes (*d*) were calculated according to Cohen’s method, in which the magnitude of the effect was classified as small (0.20–0.49), medium (0.50–0.79) or large (0.8) [56]. The α level was set at 0.05 for all tests.

## 3. Results

### 3.1. Demographic and Clinical Data on Participants

**Figure 1:**
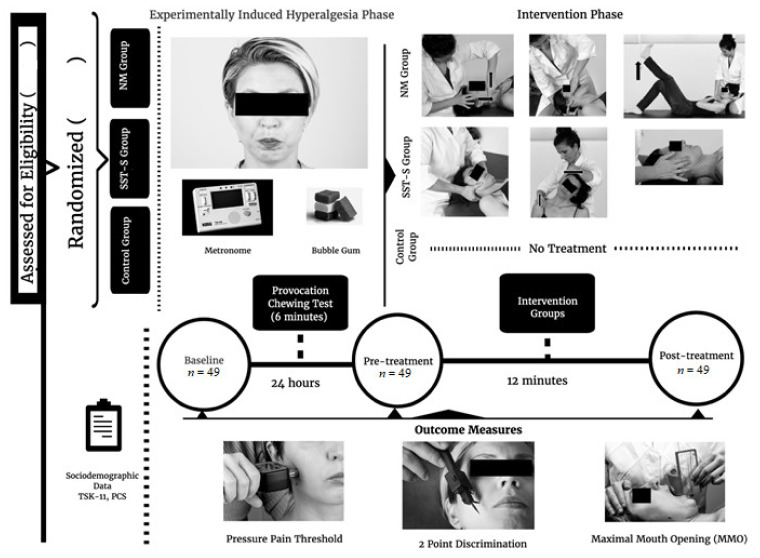
Flow diagram and procedure.

A total of 60 subjects were screened for eligibility. Forty-nine subjects between the age of 18 and 65 years old (mean = 39.06, SD = 14.99), 30 females and 19 males, with LMTrP in the masseter muscle, were included in this study, and they have been categorised into three groups. No participants dropped out during the study, and no adverse events occurred during the test. A flow diagram is provided in Figure 1. Baseline characteristics of socio-demographic, psychological and sensory-motor variables of the sample are summarised in Table 1. The statistics acquired showed no significant differences among the three groups in relation to socio-demographic variables (p > 0.05). There were no significant differences between groups for TSK-11 (*F* = 0.89, p = 0.42) and PCS (*F* = 0.33, p = 0.72) variables. The Shapiro–Wilk test was used to analyse the normal distribution of the variables (p > 0.05).

### 3.2. Pressure Pain Thresholds over Craniofacial and Cervical Muscles

**Figure 2:**
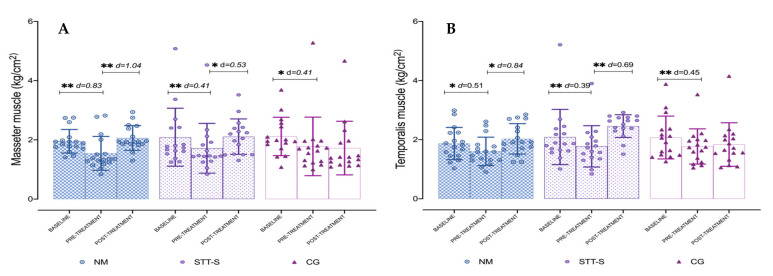
Comparison of Pressure Pain Threshold results for each of the treatment groups, at each of the measure time-points. (**A**) Masseter muscle. (**B**) Temporalis muscle. Columns represent the mean and error bars represent standard deviation. NM, neural mobilisation. STT-S, soft-tissue techniques and stretching. CG, control group. * p < 0.05; ** p < 0.001.

**Figure 3:**
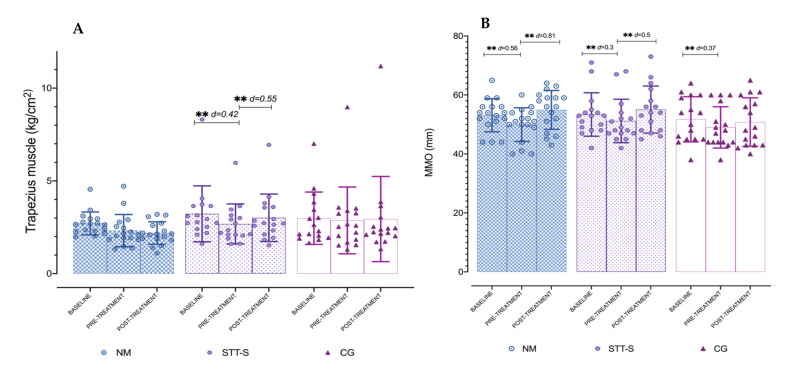
(**A**) Comparison of pressure pain threshold results of the upper trapezius muscle for each of the treatment groups, at each of the measure time-points. (**B**) Comparison of Maximal Mouth Opening for each of the treatment groups, at each of the measure time-points. Columns represent the mean and error bars represent standard deviation. NM, neural mobilisation. STT-S, soft-tissue techniques and stretching. CG, control group. ** p < 0.001.

The ANOVA revealed that significant differences were present for time × group interaction for masseter (*F* = 2.83, p = 0.029, *ηp*^2^ = 0.11), temporalis (F = 2.79, p = 0.031, *ηp*^2^ = 0.11), and trapezius (*F* = 2.62, p = 0.040, *ηp*^2^ = 0.10). The post hoc analysis showed significant differences in the comparison between baseline and pre-treatment (p < 0.01) for NM, STT-S and CG for masseter and temporalis, and in the comparison between pre-treatment and post-treatment only for NM and STT-S (p < 0.05) also for master and temporalis. For trapezius muscle, only differences were found for STT-S in both comparisons mentioned (p < 0.01). However, comparisons between baseline versus post-treatment period were not significant (p > 0.05) for any muscle. In most of the post-treatment comparisons the effect sizes were large in the NM group (*d* > 0.8), and moderate in the STT-S group (*d* = 0.5 to 0.79). The results for PPTs measured at masseter and temporalis are presented in Table 2 and Figure 2A,B; and PPTs for trapezius are represented in Figure 3A. The percentage of change is presented in Table 3. The PPTs measured at suboccipital muscles did not show significant differences (*F* = 0.88, p = 0.47, *ηp*^2^ = 0.03). The ANOVA revealed no significant differences for group interaction for any of the assessed muscles: masseter (*F* = 0.153, p = 0.859, *ηp*^2^ = 0.007), temporalis (F = 1.17, p = 0.319, *ηp*^2^ = 0.05) and trapezius (*F* = 0.872, p = 0.425, *ηp*^2^ = 0.037).

### 3.3. Maximum Mouth Opening

The ANOVA revealed significant differences for time × group interaction (*F* = 2.50, p = 0.48, *ηp*^2^ = 0.10). The post hoc analysis showed significant differences in the comparison between baseline and pre-treatment period (p < 0.01) for NM, STT-S and CG with a small effect size (*d* = 0.30 to 0.56). It also showed significant differences in the comparison between pre-treatment and post-treatment period (p < 0.01) for NM, with a large effect size (*d* = 0.81), and for STT-S, with a small effect size (*d* = 0.50). On the other hand, there were no significant differences in the comparison between baseline and post-treatment period (p > 0.05) in the NM, STT-S and CG. Descriptive data and post hoc results are shown in Figure 3B. The percentage of change is presented in Table 3. The ANOVA revealed no significant differences for group interaction for MMO (*F* = 0.7, p = 0.502, *ηp*^2^ = 0.03).

### 3.4. Two-Point Discrimination over Trigeminal and Cervical Areas

The TPD was measured at V1, V2, and cervical, but there were no significant differences in any of them, (*F* = 0.70, p = 0.58, *ηp*2 = 0.03), (*F* = 0.24, p = 0.91, *ηp*^2^ = 0.01) and (*F* = 0.501, p = 0.73, *ηp*^2^ = 0.021) respectively, nor differences between groups at V1 (*F* = 2.83, p = 0.029, *ηp*^2^ = 0.11), V2 (*F* = 2.83, p = 0.029, *ηp*^2^ = 0.11), and cervical (*F* = 2.83, p = 0.029, *ηp*^2^ = 0.11).

## 4. Discussion

The main purpose of this research was to compare the effects of the NM and STT-S on trigeminal sensory-motor variables of asymptomatic subjects who presented LMTrPs after the induction of masticatory hyperalgesia with a provocation chewing test. Significant improvements for craniofacial muscles PPTs and MMO were reached immediately after the therapeutic interventions of NM and STT-S on asymptomatic subjects with LMTrPs. Significant improvements were obtained for trapezius PPT only with the STT-S intervention. However, there were no immediate changes in TPD after the interventions. Following are listed the points that make this research novel: 1. To our knowledge this is the first clinical trial where an intervention using neural mobilisations directed to the craniofacial structures is used and compared to other manual therapy interventions. 2. Among the neural mobilisations two of them were intended to influence more specifically over the craniofacial region. 3. Immediate changes were obtained for sensory and motor outcomes.

The findings of reduced PPTs and MMO after the provocation chewing test indicates that it generated the desired effect over the masticatory muscles, suggesting that it can produce short-term hyperalgesia with the test in asymptomatic subjects with LMTrPs [57]. The intervention was established to generate DOMS on the masticatory muscles. Nonetheless, the physiology of the DOMS is discussed and controversial. Sonkodi et al. expose a novel explanation that associates them to a neural axonopathy due to the compression of the nerve endings in the muscle spindle when repetitive eccentric contractions are performed [58].

The results of the muscle sensitivity assessment showed a significant increase in the PPTs of the craniocervical muscles with both therapeutic interventions. The reached effect size was large for the NM intervention, while moderate for the STT-S intervention. However, it did not reach the MDC. Our outcomes agree with those of previous studies. For example, Oliveira-Campelo et al. [18] also assessed PPTs in the masticatory muscles, suggesting an improvement of the masseter and temporalis PPTs after the application of an atlanto-occipital joint thrust manipulation. Nevertheless, there was an improvement in the temporalis PTT but not in the masseter PPT for the STT group [18].

Regarding MMO, our results have demonstrated a significant increase after 12 min of treatment with NM and large effect size, or with STT-S and moderate effect size, surpassing both interventions the MDC. These findings are in line with those reported by Oliveira-Campelo et al. [18] who compared an atlanto-occipital joint thrust manipulation versus soft-tissue techniques (STT) on asymptomatic subjects with LMTrPs. This research found an increase in the MMO after both interventions. However, the results of the interventions were small and more studies were needed to elucidate the clinical relevance [18]. For this reason, we believe that our study offers some important findings, which strengthens and expands the previous evidence.

There are few studies evaluating the effects of manual techniques over LMTrPs. One of them, in which a cervical thrust manipulation was tested, reports a trend towards an increase in PPTs of the trapezius muscle of asymptomatic subjects [59]. Likewise, Aguilera et al. [8] and Sarrafzadeh et al. [60] have compared an STT based on ischemic compression (IC) versus ultrasound (US). They found contradictory results, no differences between both interventions or greater effects for the IC at increasing PPTs, respectively. Moreover, previous studies have also demonstrated improvements in PPTs after eight sessions of IC [61]. In the present research, the trapezius PPTs were only influenced by the SST-S intervention, with an effect of moderate size. It is clear that the present data regarding STT agree with previous literature where hypoalgesic effects are observed.

Moreover, herein it is observed that despite there being no significant differences among groups, the effect sizes are greater in the NM group. In the same line, Campa-Moran et al. [62] compared the effects of three interventions, (1) orthopaedic manual physical therapy (OMPT) including NM and articular mobilisations, (2) STT, and (3) dry needling (DN) on patients with myofascial chronic neck pain. They obtained immediate effects at reducing neck pain intensity for the STT and OMPT groups, being greater than the effects in the last one that included the NM. Furthermore, the OMPT group experienced a greater increase in PPTs in the trapezius, a decrease in pain catastrophising, and an increase in cervical ROM. Those results suggest that OMPT had a greater hypoalgesic effect than SST and DN. Although we attained good results with both interventions, NM and STT-S, our results contrast with those of Campa-Moran et al., because our study did not evidence differences between both techniques. Nevertheless, we cannot forget that Campa-Moran et al. studied chronic neck pain patients. Putting together previous data and the present data, the authors hypothesise that the addition of NM to other interventions may increase or enhance the hypoalgesic effects of the treatment.

The effects of NM reached larger effect sizes compared to the STT-S in the masticatory muscles for the PPTs. However, only the STT-S obtained a decrease in the hypersensitivity of the trapezius, but the NM did not show differences on the trapezius PPTs. The explanation may rely on the nature of the interventions themselves. The STT-S included techniques directly performed on the trapezius muscle; by contrast, the NM included specific mobilisations of the craniofacial nerves and two general techniques, which may not have had a great influence over the neck muscles as the STT-S. In this line, the effect size obtained by the NM for MMO was large and moderate for the STT-S. The present research obtained positive results for both techniques, but, in general, NM showed greater effect sizes.

NMs have been studied for many years, but the interest in this type of technique has increased in recent years. Similar to the present research, it has been demonstrated that NM may be useful for reducing pain due to DOMS in healthy adults [63]. Furthermore, it has recently been shown that NM may influence the motor performance of healthy subjects improving the height of a vertical jump [26]. It is undeniable that the special interest of NM relapses on the possible effects of these techniques on different neuromusculoskeletal conditions. Bassoon et al. highlight in their systematic review and meta-analysis the effectiveness of NM for back and neck pain, stipulating the slump, SLR, and cervical lateral glide mobilisations as the most effective techniques, while evidence of the usefulness of neurodynamics on other anatomical areas is still lacking [64], including the craniofacial region.

In clinical practice, it is frequent to use a combination of several manual techniques, and the inclusion of the NM to other OMPT or SST techniques has also been studied among the craniofacial region [65]. It has been shown that the addition of NM to the OMT techniques may obtain greater therapeutic effects of pain reduction among cervical radiculopathy patients [27,66,67]. It is theorised that NM may restore nerve homeostasis by mobilisation (passive or active) of the nerves and surrounding structures of the nervous system [24]. Animal and human studies revealed that NM improves intraneural fluid dispersion, reduces intraneural oedema, reduces mechanical and thermal hyperalgesia, and decreases temporal summation [29,68,69,70,71].

### 4.1. Limitations

The first limitation to consider is that the study only measured immediate and short-term effects. Future studies should incorporate medium and long-term interventions. The sample included a lower proportion of men than women. However, the results showed no significant differences in scores between the genders. In addition, psychosocial variables of fear of movement and pain catastrophism were only assessed as control variables. It would be interesting to investigate its influence after the intervention. In addition, the effects obtained in the present research were not the strongest, which may be justified due to the sample was composed of asymptomatic subjects who did not present pain but only LMTrP. The authors hypothesise that the results would have obtained greater differences in patients presenting with active MTrP or spontaneous pain. In this line, all the participants had LMTrP at least at the masseter muscles, but the number and specific location of the LMTrP were not registered. The sample size calculation was based on the MDC for PPT at the trapezius muscle because at that time, and to our knowledge, the MDC for masseter muscle was still not published. We now have calculated it based on the data obtained by Costa et al. [72] at the masseter muscle, and the sample size would have been of 20 participants (SD = 0.51 and MDC = 0.91). In addition, another limitation was that the treatment and assessment were performed only on the right side, which may have also contributed to the small effects obtained. Another issue is that the technique used to perform the trigeminocervical neural mobilisation could also have had its effect produced by the upper cervical mobilisation itself. We need to explain one more limitation regarding ethical reasons that made it impossible to perform a double-blind trial because the participants had to be aware they were being randomised into three possible interventions. Finally, the participants were aware that they would be randomised into three possible interventions due to the ethics committee’ requirements.

### 4.2. Clinical Implications

The LMTrP are present in healthy subjects and patients with pain together with the presence of active MTrP. The ability of the LMTrP to become active has also been described. It is interesting to investigate new approaches to manage these LMTrP that could also be extrapolated to the active MTrP. The results herein show that the NM and STT-S techniques could improve sensory and motor variables of the craniofacial system. These findings lead us to reflect on the importance of including these treatments in the clinic for specific assessment of the craniofacial region in patients with musculoskeletal pain.

We observed that STT-S had increased pain-free MMO and PPTs. For this reason, we propose that intermittent compression of the LMTrPs combined with stretching and soft-tissue mobilisation could have better results than using intermittent compression of the MTrPs in isolation, but this should be confirmed in future research. Moreover, this study has shown that an alternative treatment for patients with musculoskeletal pain could be used. We assume that NM could be beneficial in the treatment of patients who suffer a process of central sensitisation with allodynia, and the craniofacial area cannot be touched. This could be a key point in the management of pain and motor rehabilitation in patients with chronic musculoskeletal pain. Lastly, we must highlight that our results contribute to strengthening the current evidence on the beneficial effects of OMPT for patients with musculoskeletal pain.

## 5. Conclusions

With both NM and STT-S techniques, we could influence motor and sensory variables in asymptomatic subjects with LMTrPs and an experimentally masticatory induced hyperalgesia. Both techniques increased MMO and PPTs in the short term, but there were no differences between the NM and STT-S groups. These beneficial effects lead us to consider the importance of including these methods in clinical practice. However, future research should monitor the long-term effects.

## Figures and Tables

**Table 1:** Demographic and clinical data of three groups at the beginning of the study.

Measure	NM (*n* = 17)	STT-S (*n* = 16)	CG (*n* = 16)	95% CI	p Value
Age	41.7 ± 15.0	34.9 ± 16.0	44.0 ± 3.5	(34.75 to 43.37)	0.39
Gender					0.16
Male	4 (23.5%)	9 (56.3%)	6 (37.5%)		
Female	13 (76.5%)	7 (43.8%)	10 (62.5%)		
Level of studies					0.54
None	1 (5.9%)	0 (0.0%)	0 (0.0%)		
Elementary	0 (0.0%)	1 (6.3%)	0 (0.0%)		
High school	2 (11.8%)	1 (6.3%)	3 (18.8%)		
University studies	14 (82.4%)	14 (87.5%)	13 (81.3%)		
Marital status					0.78
Single	6 (35.3%)	8 (50.0%)	4 (25.0%)		
Married	8 (47.1%)	4 (25.0%)	8 (50.0%)		
Widower	0 (0.0%)	1 (6.3%)	1 (6.3%)		
Stable partner	1 (5.9%)	2 (12.5%)	1 (6.3%)		
Divorced	2 (11.8%)	1 (6.3%)	2 (12.5%)		
Employment situation					0.19
Employed/home maker	11 (64.7%)	7 (43.8%)	13 (81.3%)		
Not working	6 (35.3%)	8 (50.0%)	3 (18.8%)		
Retired	0 (0.0%)	1 (6.3%)	0 (0.0%)		
PCS (0–54 points)	9 (4, 13)	6 (5, 18.5)	7.5 (4, 13.75)		0.88
TSK-11 (0–44 points)	20 (18–25)	26 (19.25–30.75)	21.5 (17.25–27.75)		0.35

Abbreviations: NM, neural mobilisation; STT, soft-tissue techniques; S, stretching; CG, control group; PCS, pain catastrophising scale; TSK-11, Tampa scale for kinesiophobia.

**Table 2:** Comparison of changes in PPT over time for each treatment group.

Measure	Groups	Mean ± SD			Mean Difference (95% CI); p -Value; Effect Size (*d*)
		Baseline	Pre-Treatment	Post-Treatment	(a) Baseline vs. Pre-Treatment (b) Baseline vs. Post-Treatment (c) Pre-Treatment vs. Post-Treatment
PPT Masseter	NM	1.95 ± 0.40	1.54 ± 0.57	2.06 ± 0.42	(a) 0.41 (0.13 to 0.69); p = 0.002; *d* = 0.83 (b) −0.11 (−0.50 to 0.28); p > 0.05; *d* = 0.27 (c) −0.52 (−0.86 to −0.19); p = 0.01; *d* = 1.04
	STT-S	2.09 ± 0.98	1.72 ± 0.84	2.11 ± 0.60	(a) 0.37 (0.08 to 0.66); p = 0.008; *d* = 0.41 (b) −0.02 ( −0.42 to 0.38); p > 0.05; *d* = 0.02 (c) −0.39 (−0.74 to −0.05); p = 0.022; *d* = 0.53
	CG	2.12 ± 0.65	1.78 ± 0.99	1.72 ± 0.91	(a) 0.34 (0.05 to 0.63); p = 0.018; *d* = 0.41 (b) 0.39 (−0.01 to 0.80); p > 0.05; *d* = 0.51 (c) 0.06 (−0.29 to 0.40); p > 0.05; *d* = 0.06
PPT Temporalis	NM	1.87 ± 0.54	1.61 ± 0.48	2.03 ± 0.52	(a) 0.26 (0.05 to 0.47); p = 0.01; *d* = 0.51 (b) −0.16 (−0.61 to 0.30); p > 0.05; *d* = 0.30 (c) −0.42 (−0.81 to −0.03); p = 0.029; *d* = 0.84
	STT-S	2.09 ± 0.93	1.77 ± 0.70	2.16 ± 0.38	(a) 0.32 (0.10 to 0.53); p = 0.002; *d* = 0.39 (b) −0.37 (−0.84 to 0.10); p > 0.05; *d* = 0.52 (c) −0.39 (−1.08 to −0.29); p < 0.001; *d* = 0.69
	CG	2.07 ± 0.72	1.77 ± 0.60	1.83 ± 0.74	(a) 0.30 (0.09 to 0.52); p = 0.003; *d* = 0.45 (b) 0.24 (−0.23 to 0.71); p > 0.05; *d* = 0.33 (c) −0.07 (−0.46 to 0.33); p > 0.05; *d* = 0.09

Abbreviations: NM, neural mobilisation; STT, soft-tissue techniques; S, stretching; CG, control group; PPT, pressure pain threshold (kg/cm^2^); SD, standard deviation; 95% CI, 95% confidence interval.

**Table 3:** Changes of the measured outcomes in percentages.

	Groups								
Measure	**NM**			**STT**-S			**CG**		
	**% of Change**	*d*	>MDC	**% of Change**	*d*	>MDC	**% of Change**	*d*	>MDC
PPT Masseter	33.8	1.04	no	22.8	0.53	no	−0.01	0.06	no
PPT Temporalis	26.9	0.84		39	0.69		1.7	0.09	
PPT Trapezius	9.9	0.36	no	23.1	0.55	no	−6.9	0.09	no
MMO	10	0.81	yes	7.6	0.5	yes	3.7	0.23	yes

Abbreviations: NM, neural mobilisation; STT, soft-tissue techniques; S, stretching; CG, control group; PPT, pressure pain threshold; MMO, maximal mouth opening (mm). >MDC, value greater than the minimal detectable change. MDC for PPT of the masseter = 0.91 Kg/cm^2^ (Costa et al., 2019). MDC for trapezius = 1.04 Kg/cm^2^ (Walton et al., 2011). MDC for MMO = 1.79 mm (Beltran-Alacreu et al., 2014).

## Data Availability

The data presented in this study are available on request from the corresponding author.

## Abbreviations

CG	control group
CI	confidence interval
DN	dry needling
DOMS	delayed onset muscle soreness
IC	ischemic compression
LMTrPs	latent myofascial trigger points
MDC	minimal detectable change
MMO	maximum mouth opening
MTrPs	myofascial trigger points
NM	neural mobilisation
NS	nervous system
OMPT	orthopaedic manual therapy
PCS	pain catastrophising scale
PPT	pressure pain threshold
ROM	range of motion
SD	standard deviation
STT	soft-tissue technique
STT-S	soft-tissues techniques and stretching
TPD	two-point discrimination
TSK-11	Tampa scale for kinesiophobia
US	ultrasound

## Appendix A. Summary of Techniques for Neural Mobilisation Group

**Neural Thoracic Mobilisation with Wedge**		
	**Description**	**Illustration**
Patient position	Supine, with crossed legs, the right leg on top, and the left leg with knee at 90° of flexion and foot flat on the table. A wedge is placed under the upper thoracic spine.	
Therapist position	Stands on one side of the table next to the patient.	
Mobilisation technique	One hand holds the patient’s occiput and maintains a craniocervical and cervical flexion, and the other hand placed over the patient’s sternum. The mobilisation is performed alternating the following positions: (1) Pressure over the sternum, performing craniocervical and neck flexion, with flexed right knee; (2) Straighten the knee, releasing pressure over sternum. Technique performed for about 4 min.	

**Trigeminocervical Neural Mobilisation with Wedge**		
	**Description**	**Illustration**
Patient position	Supine, with bent hips and knees. The wedge is placed under the middle cervical region.	
Therapist position	Stands on one side of the table next to the patient.	
Mobilisation technique	One hand placed at the occiput and the other hand with the web space against the maxilla. The mobilisation has two alternating positions: (1) Neck flexion, craniocervical extension, and mouth opening; (2) Neutral neck position, craniocervical flexion and mouth closed. Technique performed for about 4 min.	

**Auriculotemporal Neural Mobilisation**		
	**Description**	**Illustration**
Patient position	Supine on the table.	
Therapist position	Situated at the opposite side of the joint to mobilise.	
Mobilisation technique	The cranial hand located at the patient’s forehead stabilising the head and with the fingers placed over the anterior temporal muscle. The other hand with the thumb placed over the first and second molars, and the second finger reaching the mandibular angle, and the other fingers gently folded around the body of the mandible. Alternating positions to perform the mobilisation: (1) First a distraction of the joint followed by an anterior glide; (2) Rest position of the joint and a cranial traction of the soft tissue over the temporal muscle with the hand placed over it. Technique performed for about 4 min.	

## Appendix B. Summary of Techniques for Soft-Tissue Techniques and Stretching Group

**Intermittent Compression in Latent Trigger Points**		
	**Description**	**Illustration**
Patient position	Supine on the table.	
Therapist position	Stands at the head of the table.	
Soft-tissue technique	Therapist performs an intermittent compression over the masseter, anterior temporalis and upper trapezius muscles of the right side. The therapist performs 5 cycles of 5 s compression and relaxation of 5 s.	

**Dynamic Soft-Tissue Mobilisation of Masseter Muscle**		
	**Description**	**Illustration**
Patient position	Supine on the table.	
Therapist position	Situated at the opposite side to treat.	
Soft-tissue technique	One hand placed over the frontal bone stabilising the head, the other hand placed with a pinch grip over the masseter muscle. A caudal glide movement is performed over the masseter fibres while the patient performs an active mouth opening. The technique is performed for 3.5 min, on the right side.	

**Dynamic Soft-Tissue Mobilisation of CERVICO-Craniomandibular Muscle**		
	**Description**	**Illustration**
Patient position	Supine on the table.	
Therapist position	Situated at the head of the table.	
Soft-tissue technique	Both hands are placed with the thenar eminence and thumb above the mandibular branch and masseter to perform a posterior and caudal soft-tissue mobilisation, while the other fingers are placed backwards reaching the occiput performing an anterior craniocervical roll at the same time. The technique is performed for 3.5 min.	

**Passive Stretching**		
	**Description**	**Illustration**
Patient position	Sitting in a chair with backrest and feet flat on the floor.	
Therapist position	The therapist stands behind the chair.	
Stretching technique	Upper trapezius and scalene: the therapist stretches down towards muscle insertion with the thenar of one hand while using the other grip to bend the head and cervical spine to the opposite side (left). Levator scapulae: the therapist rotates left and lateral flexes facet joints at level C1–4 to the same side by moving the head forward while with the other hand presses the shoulder. The therapist stretches for 30 s each muscle and repeats this technique two times.	
